# Efficacy of gemcitabine in combination with nanoparticle albumin‐bound paclitaxel in the treatment of recurrent ovarian cancer: A retrospective single institution review

**DOI:** 10.1002/cam4.5705

**Published:** 2023-02-21

**Authors:** Adam M. Kase, Abdel‐Ghani Azzouqa, Swapna Kochuveettil, Gerardo Colon‐Otero

**Affiliations:** ^1^ Division of Hematology and Oncology Mayo Clinic Jacksonville Florida USA; ^2^ Cancer Care Institute, Monument Health Rapid City South Dakota USA

## Abstract

**Background:**

The objective of this study was to evaluate the efficacy of gemcitabine plus nab‐paclitaxel in patients with recurrent ovarian cancer.

**Methods:**

We performed a single institution retrospective review of patients with recurrent ovarian cancer who were treated with gemcitabine plus nab‐paclitaxel from 2012 to 2018 at the Mayo Clinic in Florida.

**Results:**

Twenty patients were identified and the median PFS for patients treated with gemcitabine plus nab‐paclitaxel was 9 months (95% CI, 5.7–20.7). Overall, 17 of the 20 patients (85%) achieved a clinical benefit (complete response 5%, partial response 55%, or stable disease at 3 months 25%). For platinum‐sensitive disease and platinum‐resistant disease, the median OS were 38.7 months (95% CI, 5.8–63.1) and 31.2 months (95% CI, 12.8–51.8), respectively (*p* = 0.4306).

**Conclusion:**

This well‐tolerated regimen shows promising activity in recurrent ovarian cancer and is a viable option for patients who are intolerant to paclitaxel or carboplatin because of allergic reactions.

## Graphical abstract

In this retrospective review, 20 patients were treated with gemcitabine plus nab‐paclitaxel and the median PFS was 9 months (95% CI, 5.7–20.7). Overall, 17 of the 20 patients (85%) achieved a clinical benefit (complete response 5%, partial response 55%, or stable disease at 3 months 25%). For platinum‐sensitive disease and platinum‐resistant disease, the median OS were 38.7 months (95% CI, 5.8–63.1) and 31.2 months (95% CI, 12.8–51.8), respectively (*p* = 0.4306). This regimen is a viable and well tolerated option for patients who are intolerant to paclitaxel or carboplatin because of allergic reactions.

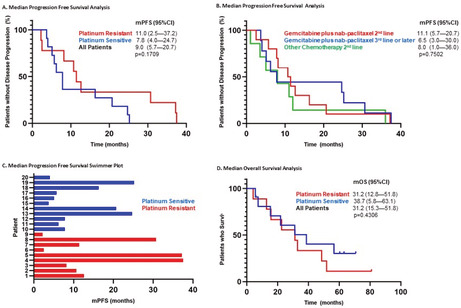

Nearly all patients with relapsed ovarian cancer will develop platinum resistance defined as relapse within 6 months after platinum‐containing therapy.^1^ These patients have poor outcomes with a median overall survival of approximately 13.3–16.6 months.^2^ The treatment for this group of patients includes single‐agent cytotoxic chemotherapy or combination therapy, with progression‐free survival ranging between 3.2 and 10.0 months. Gemcitabine has a Food and Drug Administration (FDA) approval when combined with carboplatin and an off‐label use as a single agent for the treatment of recurrent ovarian cancer. Nanoparticle albumin‐bound (nab)‐paclitaxel has an off‐label indication for recurrent ovarian cancer. Nab‐paclitaxel is frequently used in the setting of allergic reactions to paclitaxel, since almost invariably the allergic reactions to paclitaxel are the result of a reaction to the diluent. The combination of gemcitabine and nab‐paclitaxel is currently approved for the treatment of metastatic pancreatic adenocarcinoma, showing improved outcomes compared to single‐agent gemcitabine. Given the excellent tolerability of this combination and the known single‐agent activity of gemcitabine and nab‐paclitaxel against ovarian cancer, we treated patients with relapsed or refractory ovarian cancer at our institution with this combination. Mayo Clinic Institutional Review Board (IRB) approval (IRB# 17‐006677) was obtained for this study. The study was deemed exempt from written informed consent given the retrospective nature of the review. The inclusion criteria for this study included patients with relapsed or refractory ovarian cancer who were treated with gemcitabine plus nab‐paclitaxel from 2012 to 2018 at the Mayo Clinic in Florida, who were unable or unwilling to receive further platinum‐containing regimen due to platinum ineligibility. A total of 20 patients were identified as having received the combination of gemcitabine and nab‐paclitaxel and their medical records were reviewed.

Patients were treated with gemcitabine at a dose of 800 mg/m^2^ and nab‐paclitaxel at a dose of 80 mg/m^2^ on days 1, 8, 15 every 28 days. Patients were treated if their ANC was 1000 or greater and if their platelet count was 100,000 or greater. If patients were not able to receive treatment on time, their schedule was modified to days 1 and 8 every 21 days. If they were not able to tolerate this schedule, the doses were modified further to be given once every 14 days. GraphPad Prism 8.4.3 was used for descriptive and comparison statistical analysis. To analyze PFS, time to event was obtained using Kaplan–Meier method and compared using log‐rank test. Statistical significance was considered if *p* < 0.05. Confidence intervals at 95% are reported.

Twenty patients were identified as having received the combination of gemcitabine and nab‐paclitaxel. The mean age of the 20 patients who were treated with combination gemcitabine and nab‐paclitaxel was 66.2 with a standard deviation 9.5. Additional demographic information is available within Table 1. Only one patient had clear cell histology while the remaining 19 patients (95%) had serous ovarian cancer. One patient had low‐grade serous carcinoma and 18 (90%) had high grade serous ovarian cancer. 75% of patients had stage III or IV disease at time of initial diagnosis. Of the 20 patients, 45% (*n* = 9) had platinum‐resistant disease. The median previous lines of therapy were 2 with 50%, 35%, 5%, and 10% of patients previously treated with one, two, three, or four plus lines of therapy, respectively. Nine patients (45%) were previously treated with gemcitabine and two patients (10%) were previously treated with nab‐paclitaxel.

**TABLE 1:** Distribution of baseline characteristics.

Characteristic (*n* = 20)	Gemcitabine with nab‐paclitaxel, *n* (%)
Age, years	
Mean (stand deviation)	66.2 (9.5)
Race	
White	18 (90)
Black	0
Hispanic	0
Asian	2 (10)
Initial FIGO stage III or IV	15 (75)
Histology at diagnosis	
High‐grade serous	18 (90)
Clear cell	1 (5)
Low‐grade serous	1 (5)
Previous lines of therapy	
1	10 (50)
2	7 (35)
3	1 (5)
4 or more	2 (10)
Previous exposure to gemcitabine	9 (45)
Previous exposure to nab‐paclitaxel	2 (10)
Platinum resistant	9 (45)
PFS while on platinum	
≤ 6 months	5
7–12 months	9
13–18 months	3
>18 months	2
Unknown	1

One patient (5%) had a complete response, 11 patients (55%) had a partial response, and 5 patients (25%) had stable disease for at least 3 months (Figure 1). The ORR for patients with a platinum‐free interval of <12 months was 57.1% compared with 80% in patients with a PFI ≥12 months. Of the 12 patients where a response was observed with decrease in tumor size, four patients (26%) were switched to maintenance therapy gemcitabine with nab‐paclitaxel every other week. Overall, 17 of 20 patients (85%) achieved a clinical benefit (complete remission, partial remission, or stable disease for at least 3 months).

**FIGURE 1:**
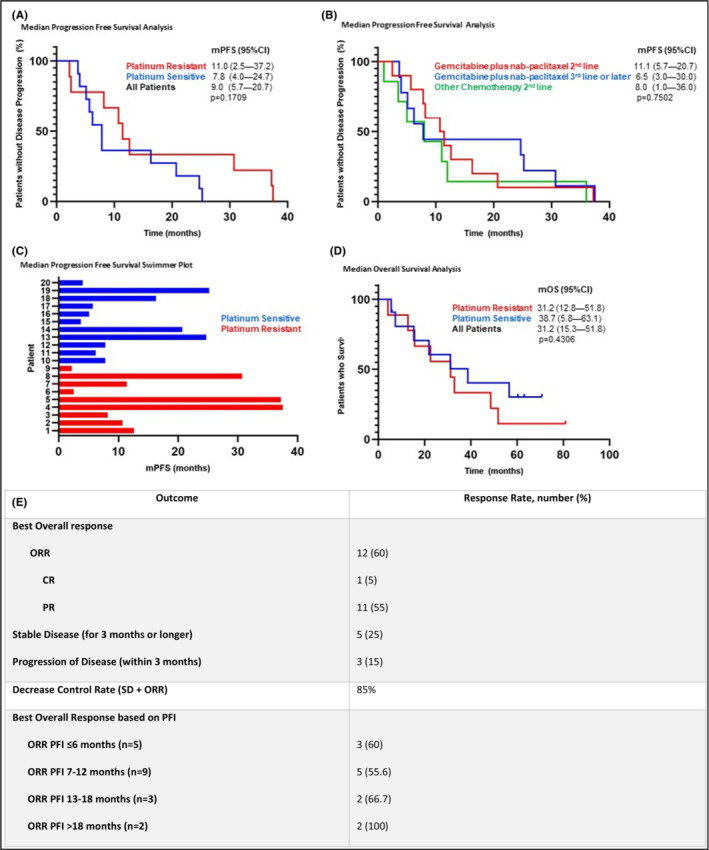
Outcome analysis. (A) Kaplan–Meier estimates comparing mPFS of patients with platinum‐sensitive and platinum‐resistant ovarian cancer who were treated with gemcitabine with nab‐paclitaxel. (B) Comparison of mPFS between patients who received gemcitabine with nab‐paclitaxel in the second‐line setting or third‐line setting, with additional comparison to patients who received other chemotherapy in the second‐line setting. (C) Swimmer plot showing each patient in the study with correlated mPFS when treated with gemcitabine with nab‐paclitaxel. (D) Comparison of mOS of patients with platinum‐sensitive and platinum‐resistant ovarian cancer who were treated with gemcitabine with nab‐paclitaxel. (E) Clinical response rate. mPFS, median progression‐free survival; SD, stable disease; PD, progression of disease at first scan; ORR, overall response rate; CR, complete response; PR, partial response; PFI, platinum‐free interval.

The median progression‐free survival (mPFS) was 9.0 months (95% CI, 5.7–20.7) (Figure 1). The mPFS was longer when gemcitabine with nab‐paclitaxel was given in the second‐line setting compared with the third line (11.1 vs. 6.5 months*, p* = 0.7502). Compared with other chemotherapy agents used in the second‐line setting in this group of patients, gemcitabine with nab‐paclitaxel had a longer mPFS (11.1 vs. 8.0 months, *p* = 0.7502). The median overall survival was 31.2 months (95% CI, 15.3–51.8) (Figure 1). For platinum‐sensitive disease and platinum‐resistant disease the mOS was 38.7 (95% CI, 5.8–63.1) and 31.2 months (95% CI, 12.8–51.8), respectively (*p* = 0.4306).

Eight of the 20 patients (40%) required a dose reduction with five patients (20%) having grade 3 neutropenia. For patients with recurrent cytopenias, the regimen was adjusted to every 2 weeks or given on days 1 and days 8 every 21 days. Two of the 20 patients (10%) had a history of infusion reaction to paclitaxel, and both patients tolerated nab‐paclitaxel without an infusion reaction.

In patients with recurrent ovarian cancer, the designation of platinum sensitive and platinum resistance is utilized to guide therapy. More recently, the definition of platinum‐resistant disease is being challenged to instead consider platinum resistance as those who progress during platinum‐based therapy or in those with symptomatic relapse soon after completing platinum therapy, since patients with platinum‐free interval (PFI) of less than 6 months can still achieve a response to platinum agents.^3^ For patients with recurrent ovarian cancer that are not platinum eligible and receive single agent or combinatory therapy, the average ORR, mPFS, and mOS are 23.5%, 4.4 months, and 13.5 months, respectively.

Nab‐paclitaxel has an off‐label use indication for recurrent ovarian cancer given at 260 mg/m^2^ on Day 1 of a 21‐day cycle or 100 mg/m^2^ on Days 1, 8, and 15 of a 28‐day cycle. In a phase II open label study, patients who progressed after one line of platinum containing therapy were treated with nab‐paclitaxel alone at 260 mg/m^2^ and achieved a Response Evaluation Criteria in Solid Tumors (RECIST) based ORR of 45% (1 CR and 13 PR) and mPFS of 8.5 months.^4^ In the phase II trial of nab‐paclitaxel at 100 mg/m^2^, the ORR was 23% (1 CR and 10 PR) and 36% (*n* = 17) had stable disease in addition to a mPFS of 4.5 months and mOS of 17.4 months.^5^ Gemcitabine has an FDA approval when combined with carboplatin in patients with platinum sensitive recurrent ovarian cancer in addition to an off‐label use as a single agent. Pfisterer et al showed that carboplatin plus gemcitabine resulted in an ORR and mPFS of 47.2% and 8.6 months, respectively.^6^

In the study presented here, the ORR and mPFS of gemcitabine plus nab‐paclitaxel was 60% and 9.0 months, respectively. The ORR was higher in patients with a platinum‐free interval of >12 months (80%) compared to ≤12 months (57.1%). The mPFS was 11 months in the platinum‐resistant subset. This suggests that gemcitabine plus nab‐paclitaxel is a promising treatment option for the platinum‐resistant patient population where the average ORR is 23.5% and average mPFS is around 4.4 months in previously approved treatment regimens (Figure 1). Gemcitabine with nab‐paclitaxel appears to result in a higher PFS when used in the second line (11.1 months) compared with third line (6.5 months), as expected. In addition, second‐line gemcitabine with nab‐paclitaxel results in a longer mPFS (11.1 months) versus other second‐line chemotherapy (8.0 months) used in the study patient population.

Historically, second‐line carboplatin plus paclitaxel given to patients with platinum‐sensitive disease results in an ORR of 0%, 23%, and 77% for a PFI of <6, 6–12, and >12 months, respectively.^7^ For patients with platinum sensitive recurrent disease, liposomal doxorubicin, and carboplatin results in an ORR of 35%, 28 %, and 37 % in patients with a PFI of <6, 6–12 and >12 months, respectively.^8^ In our cohort presented here, the ORR was 60%, 55.6 %, and 80%, in patients with a PFI of <6, 6–12, and >12 months, respectively. These findings suggest that gemcitabine and nab‐paclitaxel is a reasonable option in platinum‐sensitive recurrent disease, particularly in patients unable to receive platinum or paclitaxel due to allergic reactions.

Dose reductions were required in eight patients (40%) because of cytopenias. Seven of the eight patients (87.5%) who required a dose reduction still had a partial response and an mPFS of 11.7 months. This shows the tolerability of these regimen and ability to dose reduce without an impact on efficacy. If a dose reduction is required then a 20% dose reduction could be considered or changing the regimen to Day 1, Day 8 of every 21 days. After two to four cycles of treatment on Days 1, 8, and 15 of every 28 days, four patients (20%) were switched to maintenance therapy with gemcitabine and nab‐paclitaxel every other week. These patients achieved an mPFS of 31.2 months. This suggests a treatment every other week after two to six cycles could be considered to allow for better tolerability. The limitations of this study include the fact that this is a retrospective analysis of a single institution experience and the limited number of cases included in this review. Potential selection bias was addressed by including all cases treated at our institution during the study period.

Management of recurrent ovarian cancer remains challenging with low response rates and short progression‐free survival. Gemcitabine plus nab‐paclitaxel is a promising combinatory therapy in the second‐line setting and beyond for patients with platinum sensitive or resistant recurrent ovarian cancer. This treatment is well tolerated and represents an alternative treatment for patients who have recurrent disease or in patients who are intolerant to paclitaxel or carboplatin because of allergic reactions.

## AUTHOR CONTRIBUTIONS

**Adam M. Kase:** Conceptualization (supporting); data curation (supporting); formal analysis (equal); visualization (equal); writing – original draft (lead); writing – review and editing (equal). **Abdel‐Ghani Azzouqa:** Conceptualization (supporting); data curation (supporting); writing – review and editing (supporting). **Swapna Kochuveettil:** Data curation (supporting); writing – review and editing (supporting). **Gerardo Colon‐Otero:** Conceptualization (equal); formal analysis (equal); writing – original draft (equal); writing – review and editing (equal).

## FUNDING INFORMATION

No funding was received for this review.

## CONFLICT OF INTEREST STATEMENT

The authors have no conflicts of interest.

## Data Availability

The data that support the findings of this study are available from the corresponding author upon reasonable request.
